# Metagenomic analysis reveals a dynamic microbiome with diversified adaptive functions that respond to ovulation regulation in the mouse endometrium

**DOI:** 10.1186/s12864-023-09712-8

**Published:** 2023-10-13

**Authors:** Sisi Pu, Meng Wang, Jinglei Wang, Qian Zhang, Xin Ma, Rui Wang, Sijiu Yu, Libin Wang, Yangyang Pan

**Affiliations:** 1 College of Veterinary Medicine, Gansu Agricultural University, Lanzhou, Gansu China; 2 Technology and Research Center of Gansu Province for Embryonic Engineering of Bovine and Sheep & Goat, Lanzhou, Gansu China

**Keywords:** Endometrial microbes, Reproductive regulation, Supernumerary ovulation, Reproductive health, Microbial diversity

## Abstract

Understanding the microflora inhabiting the reproductive tract is important for a better understanding of female physiology and reproductive health. The endometrial fluid from mice in three reproductive stages (A: Unproductive mice; B: Postovulatory mice; C: Postpartum mice) was extracted for microbial DNA extraction and sequencing. Phenotypic and functional analyses of endometrial microbial enrichment was undertaken using LefSe. The results showed 95 genera and 134 species of microorganisms in the uteri of mice. There were differentially distributed genera, among which *Lactobacillus, Enterococcus*, and *Streptococcus* were more abundant in the endometrial fluid of mice in the unproductive group. That of mice in the postovulatory group was colonized with *Salmonella enterica* and *Campylobacter* and was mainly enriched in metabolic pathways and steroid biosynthesis. The presence of *Chlamydia, Enterococcus, Pseudomonadales, Acinetobacter*, and *Clostridium* in the endometrial fluid of postpartum mice, in addition to the enrichment of the endocrine system and the Apelin and FoxO signaling pathways, resulted in a higher number of pathogenic pathways than in the other two groups. The results showed that the microbial diversity characteristics in the endometrium of mice in different reproductive states differed and that they could be involved in the regulation of animal reproduction through metabolic pathways and steroid biosynthesis, suggesting that reproductive diseases induced by microbial diversity alterations in the regulation of animal reproduction cannot be ignored.

## Introduction

Macrogenomics is used extensively to understand the structure of microbial communities, uncover new microbial species, elucidate microbial functions, monitor environmental changes, and analyze microbial interactions [1, 2]. Bacterial vaginitis, endometriosis, and polycystic ovarian syndrome can cause changes in the microbial community of the reproductive tract, demonstrating that microbial diversity is closely linked to reproductive tract health [3–7]. Therefore, the use of macrogenomics to explore the microbial diversity of the reproductive system is important for understanding the molecular mechanisms of microbial involvement in reproductive regulation and alteration of reproductive health. In recent years, the exploration of microbial characteristics has mainly focused on the vagina [8–10]. There is consequently a shortage of studies on microbial characteristics in the uterus related to ovulation regulation. Exploring the association between microbial communities colonizing the uteri of female animals and reproductive regulation will contribute to a more comprehensive understanding of uterine health in females.

Progesterone and estrogen levels change according to the reproductive status of animals [11, 12], while microorganisms are engaged in the regulation of hormones and can trigger reproductive tract diseases. For example, changes in the percentage of microorganisms in the uteri of mice after parturition leads to a decrease in the abundance of *Firmicutes* and an increase in the abundance of *Proteobacteria*, both of which are closely associated with endometrial disease [13]. In addition, altered reproductive status in animals leads to fluctuations in hormone levels in the genital tract [14, 15]. For instance, estrogen affects the proportion of *Candida albicans* and *Escherichia coli* in the vagina [16–18]. Despite this, the regulation of microbial diversity in the mouse uterus has not yet been reported on. Therefore, observation of microflora within the uterus during reproductive regulation may contribute to an improved understanding of interactions between microorganisms and reproductive hormones. In this study, endometrial fluid samples were collected from unproductive, postovulatory, and postpartum mice to explore their uterine microbial diversity in different reproductive states and to predict the function of the dominant flora and its role in reproductive regulation and health using macrogenomic techniques.

## Methods

### Animals and samples

This study was approved by the Ethics Committee of Gansu Agricultural University, China (ethics approval file No. GSAU-Eth-VMC-2023-006). Animal experiments, including sample collection, were performed in accordance with the guidelines of the Ethics Committee of Gansu Agricultural University. Furthermore, the experimental protocol complied with local animal welfare guidelines.

The sample size was calculated using G * Power, effect size f = 0.5, α = 0.05, 1-β = 0.8, and the total sample size was calculated to be nine mice, three in each group. The test animals consisted of nine 30-day-old SPF-grade BALB/c mice, which were purchased from the Lanzhou Veterinary Research Institute, Chinese Academy of Agricultural Sciences. Their weights were between 18 and 22 g. Six unproductive female mice were randomly divided into two groups and labeled as groups A and B, respectively. Three postpartum female mice were chosen and labeled as group C. Group A and C was not treated and endometrial fluid samples were collected from mice. Immature mice from group B were injected intraperitoneally with five IU PMSG (Pregnant mare serum gonadotropin)to stimulate preovulatory follicle development followed 48 h later with five IU LH(Luteinizing hormone)to stimulate ovulation, which reference the methods described in previous studies [19–21]. Samples were collected 20 h after administration.

### Nucleic acid extraction

Genomic DNA was extracted using a HiPure Bacterial DNA Kit (Magen, Guangzhou, China) according to the manufacturer’s instructions. The OD260 / OD280 was between 1.6 and 1.8 detected by NanoDrop 2000 spectrophotometer (Thermo Fisher Scientific, Waltham, MA). The DNA samples were subjected to 1.0% agarose gel electrophoresis ( voltage: 120 V ), and then the total DNA amount of gel pores was detected by Qubit (Thermo Fisher Scientific, Waltham, MA, USA) fluorescence spectrophotometer. Sample concentration > 20ng / uL, total DNA ≥ 1 µg.

### Metagenomic libraries construction and sequencing

Extracted genomic DNA was firstly fragmented by sonication to a size of 350 bp, before being end-repaired, A-tailed, and adaptor-ligated using the NEBNext® ΜLtra™ DNA Library Prep Kit for Illumina (NEB, USA) according to the preparation protocol. DNA fragments with lengths of 300–400 bp were amplified by PCR. Finally, PCR products were purified using the AMPure XP system (Beckman Coulter, Brea, CA, USA), and the libraries were analyzed for size distribution using a 2100 Bioanalyzer (Agilent, Santa Clara, CA, USA) and quantified using real-time PCR. Genome sequencing was performed using the Illumina NovaSeq 6000 sequencer with paired-end technology (PE 150) [22].

### Data processing and bioinformatics analysis

#### Quality control

Raw data from the Illumina platform were filtered using FASTP software (version 0.18.0) [23]. Reads with ≥ 10% unidentified nucleotides (N), removing reads with ≥ 50% bases having phred quality scores ≤ 20, and those aligned to the barcode adapter were removed. After filtering, the clean reads were used for genome assembly.

#### Assembly, gene prediction and gene catalogue

Clean reads from each sample were assembled individually using MEGAHIT (version 1.1.2) [24] stepping over a k-mer range of 21–99 to generate a sample-derived assembly. Genes were predicted based on the final assembly contigs (> 500 bp) using MetaGeneMark (version 3.38) [25]. The predicted genes ≥ 300 bp in length from all samples were pooled and combined based on ≥ 95% identity and 90% reads coverage using CD-HIT (version 4.6) [26] in order to reduce the number of redundant genes for the downstream assembly step. The reads were realigned to the predicted gene using Bowtie (version 2.2.5) [27] to count read numbers. The final gene catalogue was obtained from non-redundant genes with a gene read count of > 2%.

#### Function annotations

The assembled sequences were annotated using complementary methods. Single genes were annotated using DIAMOND (version 0.9.24) [28] against proteins deposited in different protein databases, including the National Center for Biotechnology Information (NCBI) Non-Redundant Protein (Nr) database, Kyoto Encyclopedia of Genes and Genomes (KEGG), Evolutionary Genealogy of Genes, and the Unsupervised Direct Lineage Group (eggNOG).

#### Taxonomic profiling

Using Kaiju (version 1.6.3) [29], clean reads were translated into amino acid sequences, and the species annotation of reads was obtained by comparing with the Nr microbial library. Based on the number of reads on the comparison, the species abundance was obtained. We use the stacking diagram to visually display the species abundance of each sample at each level of classification, and initially present the species distribution law between samples, dominant species and other information.

#### Comparative analysis

Multivariate statistical techniques, including principal coordinate analysis (PCoA) of Bray–Curtis distances, were performed using the R vegan package (https://cran.r-project.org) and plotted using the R ggplot2 package (https://cran.r-project.org). The biomarker characteristics of each group were screened using Lefse software (version 1.0) [30].

### RNA isolation and relative quantification using quantitative real-time polymerase chain reaction

 Total RNA was extracted using E.Z.N.A.® Bacterial RNA Kit, following the manufacturer’s instructions. A Nanodrop 2,000c spectrophotometer (xThermo Fisher Scientific) was used to analyze RNA concentrations. Total RNA was reverse transcribed using a HiScript II Q RT SuperMix, and qRT-PCR was performed using the SYBR method. The 16 S universal Eubacterial primers 530 F (5′-GTGCCAGCMGCNGCGG-3′)-1100R (5′-GGGTTNCGNTCGTTG-3′) described by Dowd et al. [31], was selected and commonly used in earlier literature. Other primers used are listed in Table 1. The fold change in gene expression was determined using the ΔΔCT method. The experiments were performed in triplicate.

**Table 1:** List of primers for quantifying microbial genes from endometrial fluid samples

Primer Set	Primers	Sequence	Application
16 S rRNA	Forward	5′-GTGCCAGCMGCNGCGG-3′	qRT- PCR
	Reverse	5′-GGGTTNCGNTCGTTG-3′	
Enterococcus	Forward	5′-GGATTGCCTCAAGAAGCGACTG-3′	qRT- PCR
	Reverse	5′-AAGCGATCACTGCGTTCATAGAC-3′	
Salmonella enterica	Forward	5′-CCTGAATCACTTACCCGTCAAACTG-3′	qRT- PCR
	Reverse	5′-ACGCCATTCGCCATAATCTCAAC-3′	
Rickettsiales	Forward	5′-GTTGGTTGTACTGCTGGTCCTAAG-3′	qRT- PCR
	Reverse	5′-ACTCTTCATTACCTTGTAGCCATCC-3′	

## Results

### Libraries sequencing data and gene prediction

**Fig. 1:**
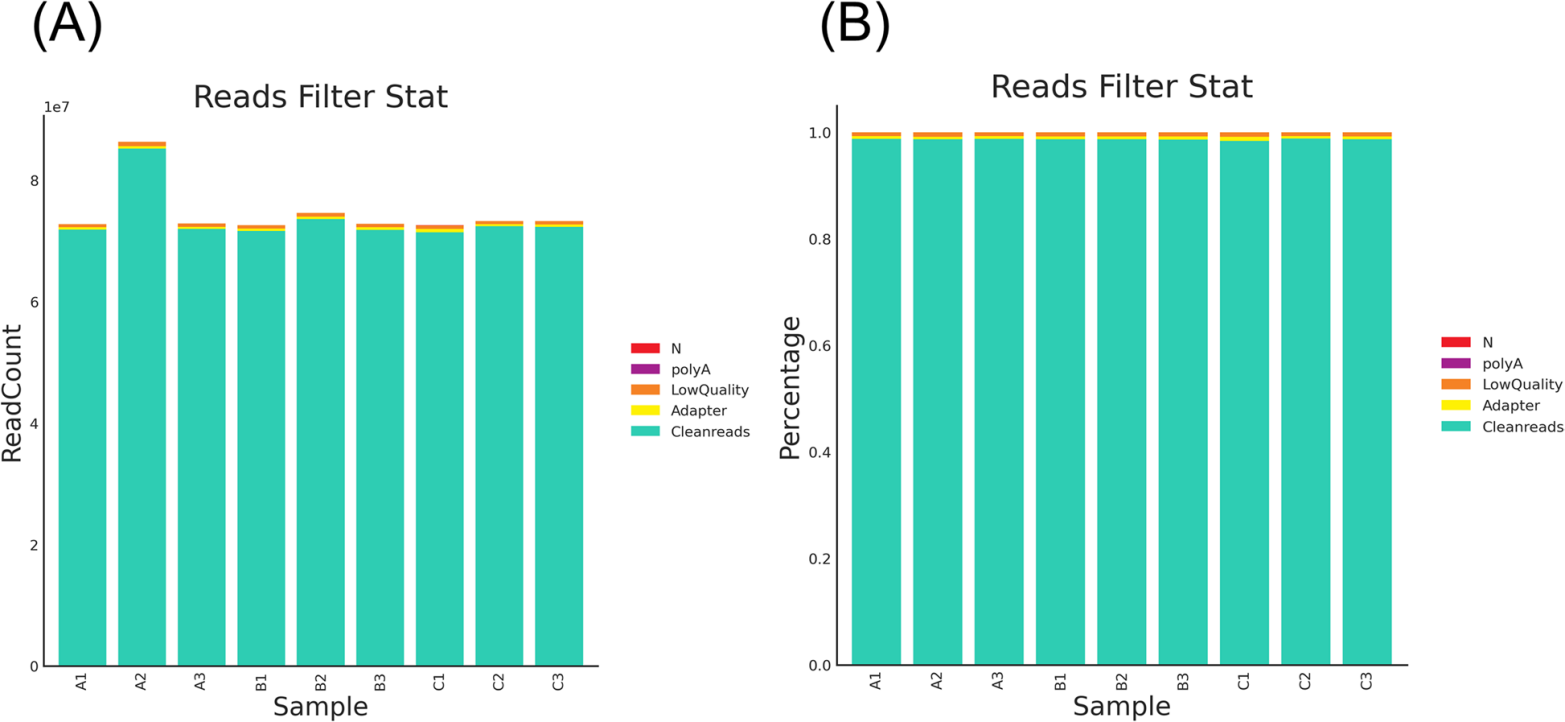
Raw data processing results of endometrial samples

**Fig. 2:**
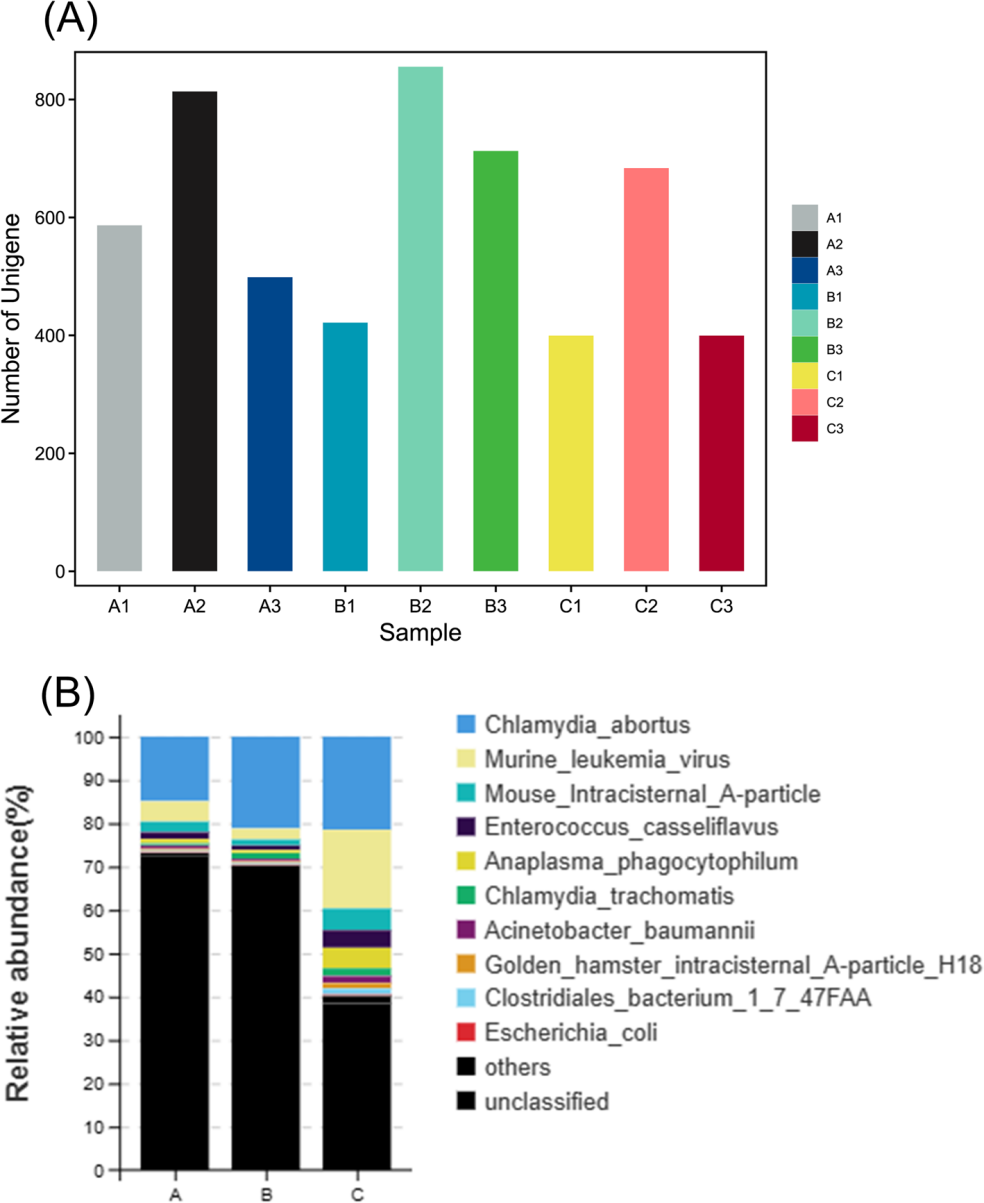
**A** Number of unigene; **B** Pathogenic microorganism distribution stack diagram

 Sequencing produced 668,687,394 raw reads. After preprocessing, 662,461,780 usable clean reads, with an average of 73,606,864 reads per sample, remained in the dataset (Fig. 1A). The average number of bases per sample before quality control was 11,144,789,900 bp and the average number of effective bases per sample after quality control was 10,881,571,595 bp. In total, 662,461,780 high-quality reads were obtained using host-sequence filtering (Fig. 1B). Finally, the effective reads were assembled using MEGAHIT software, and the average contig length of each sample was 935.5 bp. There were no significant differences between the three groups (Fig. 2A).

### Bacterial diversity and community structure of Intrauterine Microbiota

**Fig. 3:**
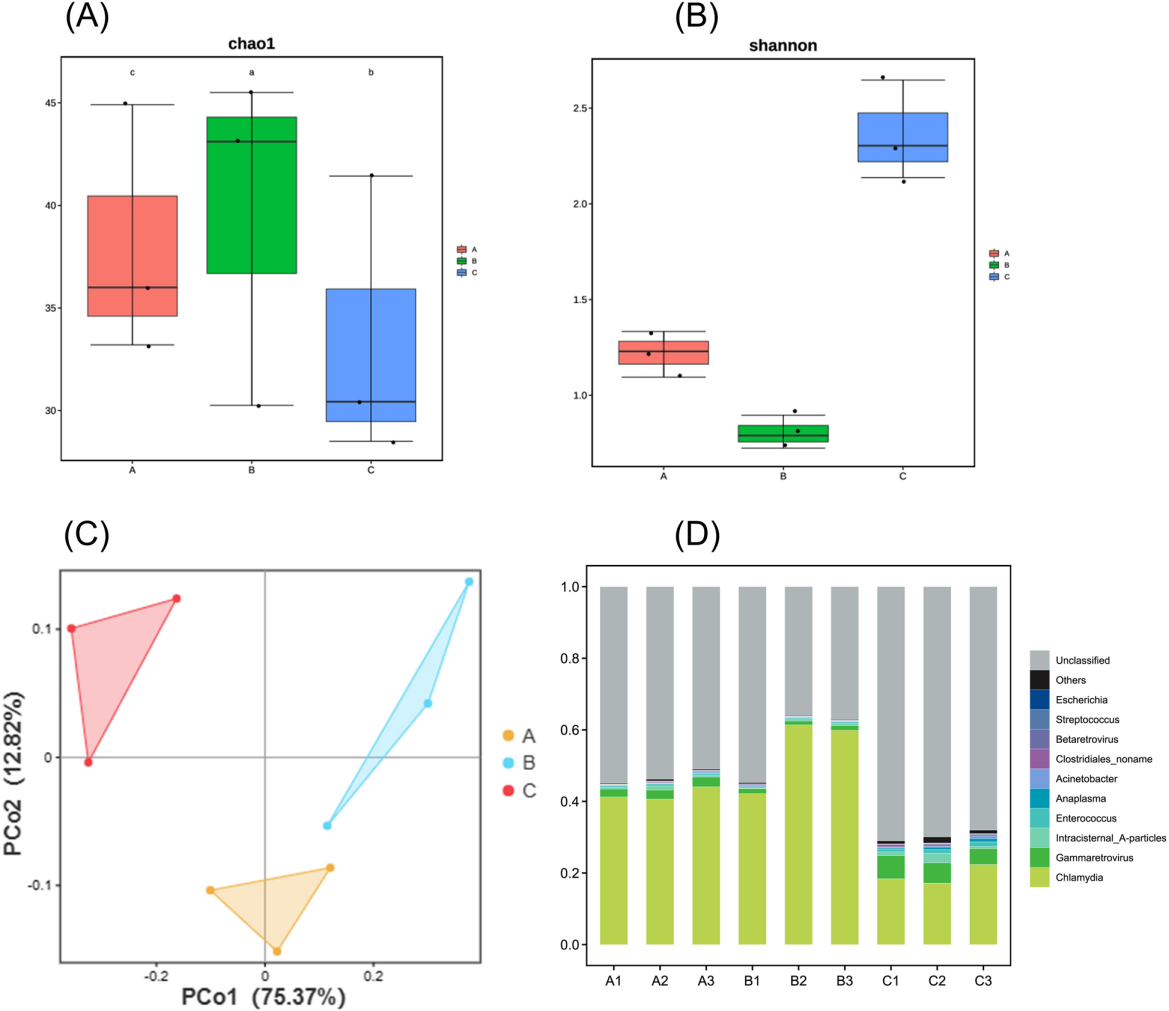
**A** Chao 1 index for three groups; **B** Shannon index for three groups; **C** Principal coordinates analysis(PCoA) plot; **D** Phylum distribution stacking diagram of mice endometrial microorganisms

 Community richness, as estimated by the Chao 1 index, was higher in group B than in groups A or C (Fig. 3A). The Shannon index, an alpha diversity estimator, was higher in group C than in groups A and B, indicating higher bacterial diversity of the endometrial microbiota in group C (Fig. 3B). Structural similarity was explored using PCoA of beta diversity analysis (Fig. 3C), which contained the first two principal coordinate axes (factors). Factor 1 accounted for 75.37% of the total variation, whereas factor 2 explained 12.82% of the variation. Clustering and ordination were correlated with the environmental variables that controlled microbial community composition. These data indicate that ovulation regulation can alter the diversity and composition of the uterine microbiota in mice.

To characterize the distribution of bacteria, the relative abundance of the endometrial microbiota was analyzed in groups A, B, and C. In total, 23 phyla, 35 classes, 55 orders, 71 families, 95 genera, and 134 species were identified. Group A comprised *Lactobacillales, Enterococcus*, and *Streptococcus*. Among the major phyla, *Proteobacteria* and *Firmicutes* were the most abundant among the three groups (Fig. 3D) and constituted the major endometrial phyla. The main orders in group C included *Enterococcus, Lactobacillales, Clostridiales, Rickettsiales, Pseudomonadales*, and *Acinetobacter. Salmonella enterica, Campylobacter*, and *Chlamydiae* were the most abundant species in group B. The main order in group A was *Lactobacillus*, which included *Enterococcus* and *Streptococcus*. Species analysis in the three groups for pathogenic microorganisms (Fig. 2B) showed that group C had the highest proportions of pathogenicity, including *Chlamydia abortus, Murine leukemia virus, Mouse intracisternal A particle, Enterococcus casseliflavus, Acinetobacter baumannii, Golden hamster intracisternal A particle H18*, and *Clostridiales bacterium 1_7_47FAA*.

### Comparison of microbial phenotypes in response to ovulation regulation

**Fig. 4:**
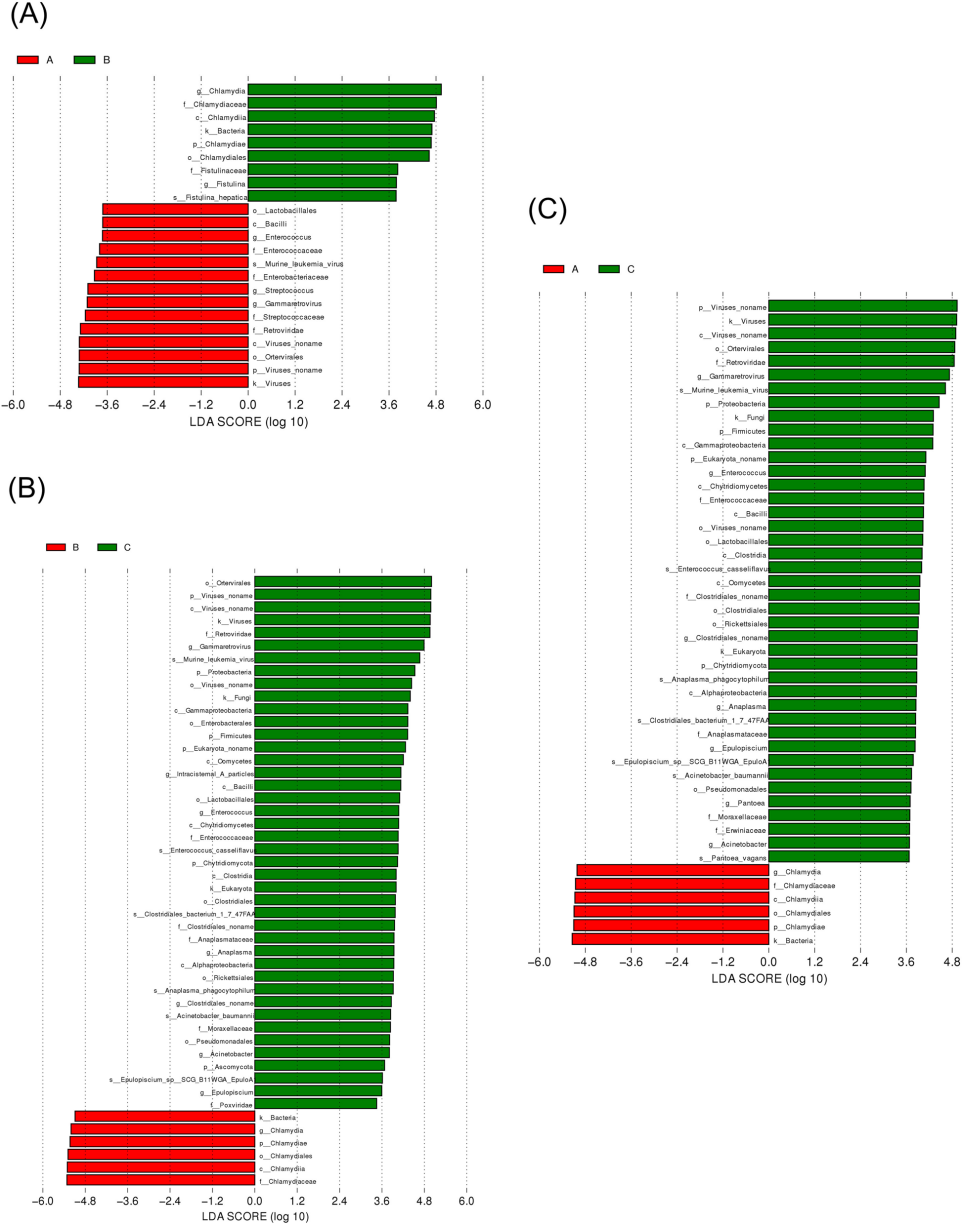
LDA score analysis of phenotypes among three groups

**Fig. 5:**
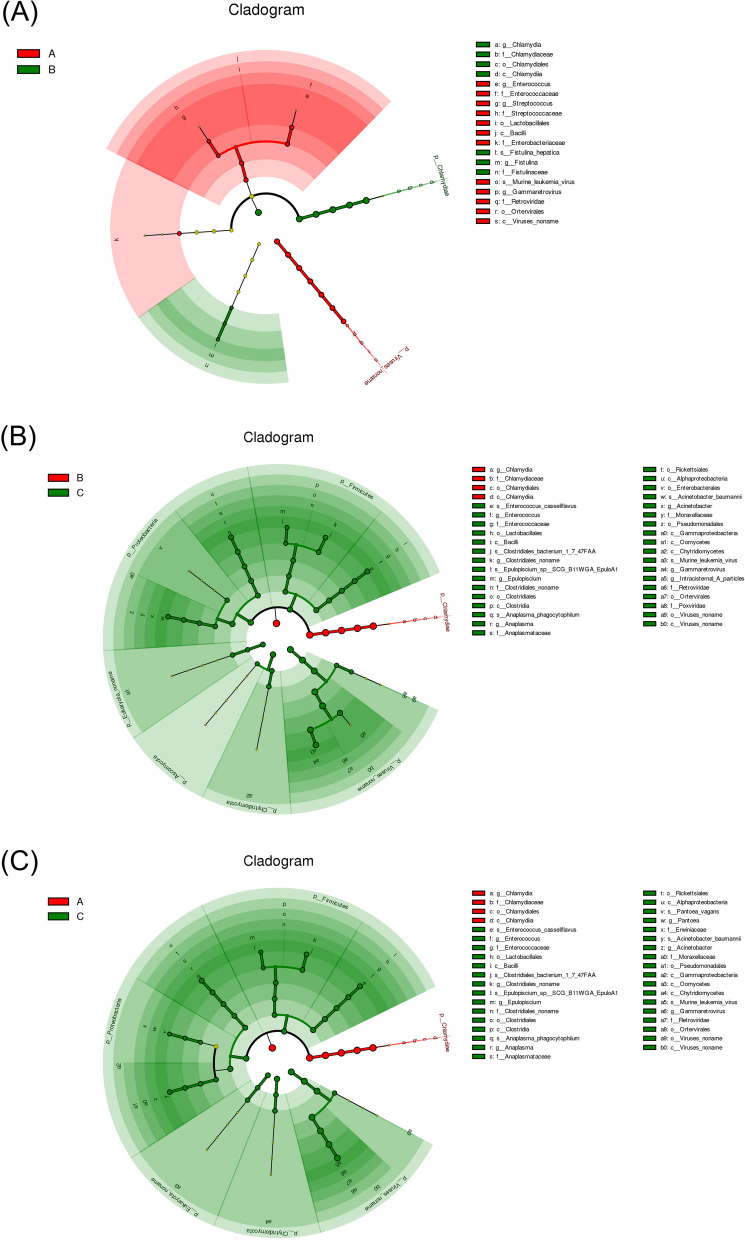
Cladogram analysis of phenotypes among three groups

 LefSe was used to identify the phylotype that most likely explained the differences between the endometrial fluid samples from groups A, B, and C. A circular cladogram was generated to show the differentially abundant taxa, and LDA coupled with effect size measurements was used to assess the effect size of the taxa (Figs. 4 and 5). Compared to group A, group B was mainly enriched in *Salmonella enterica* and *Campylobacter*. Group C consisted of *Proteobacteria* including *Pseudomonadales*, and *Fimicutes* including *Enterococcus, Lactobacillus*, and *Clostridiales*. Groups A and C were enriched in the same genus of *Firmicutes* [32]. The effect sizes and ranking of the LDA scores indicated that the differential enrichment of bacteria was owing to the different ovulation methods.

### Analysis of microbial functional differences in response to ovulation regulation

**Fig. 6:**
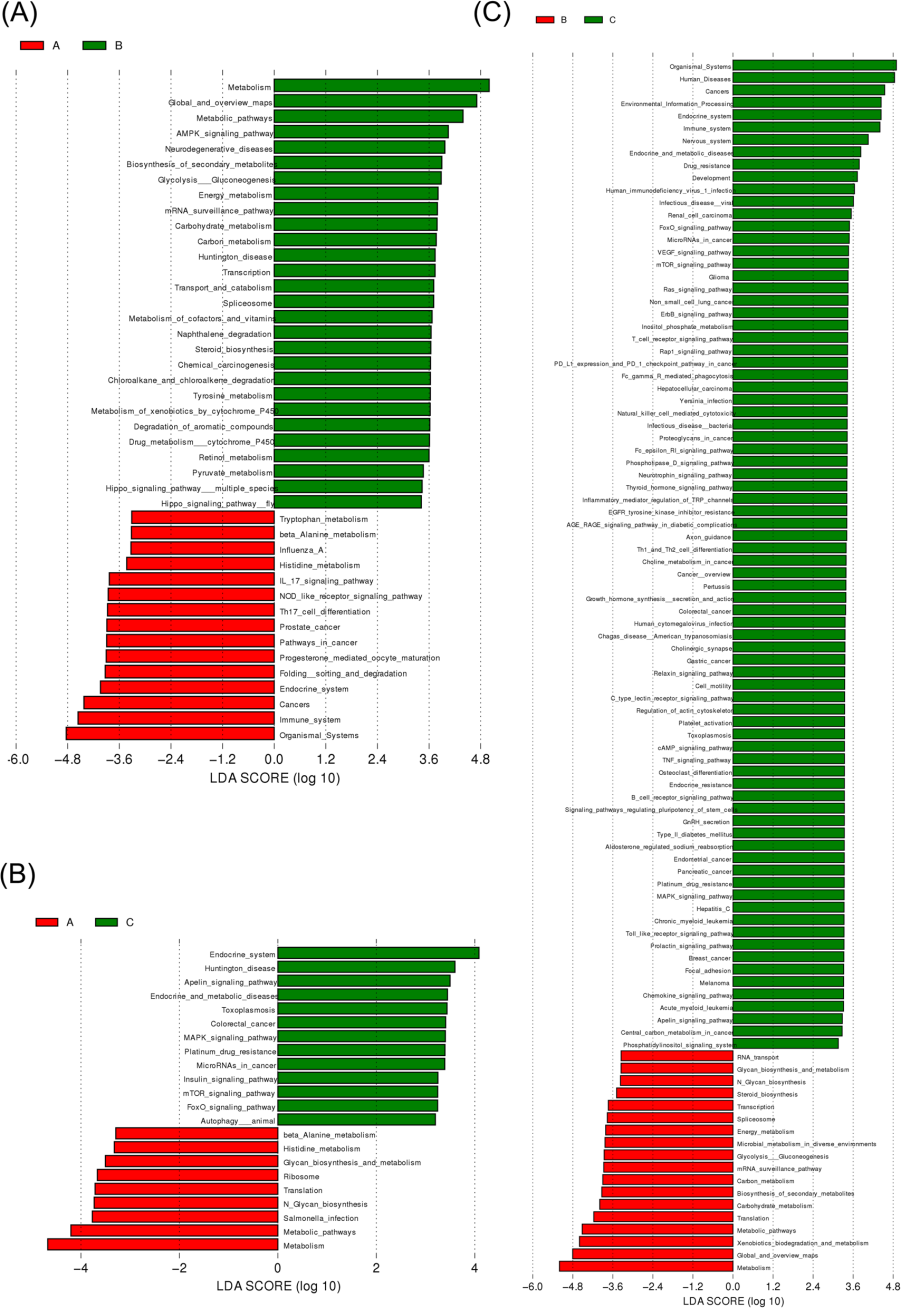
LDA score analysis of functions among three groups

**Fig. 7:**
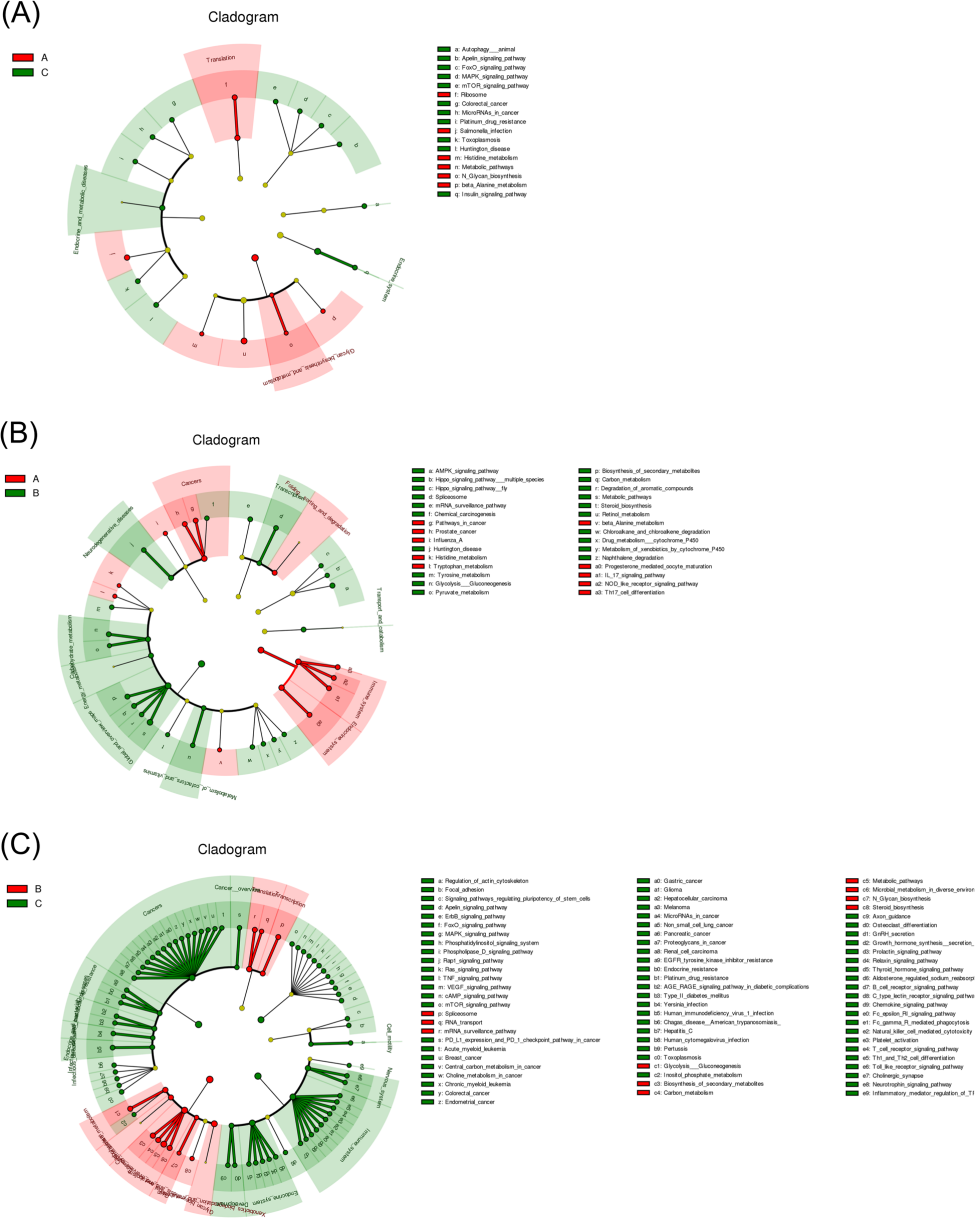
Cladogram analysis of functions among three groups

**Fig. 8:**
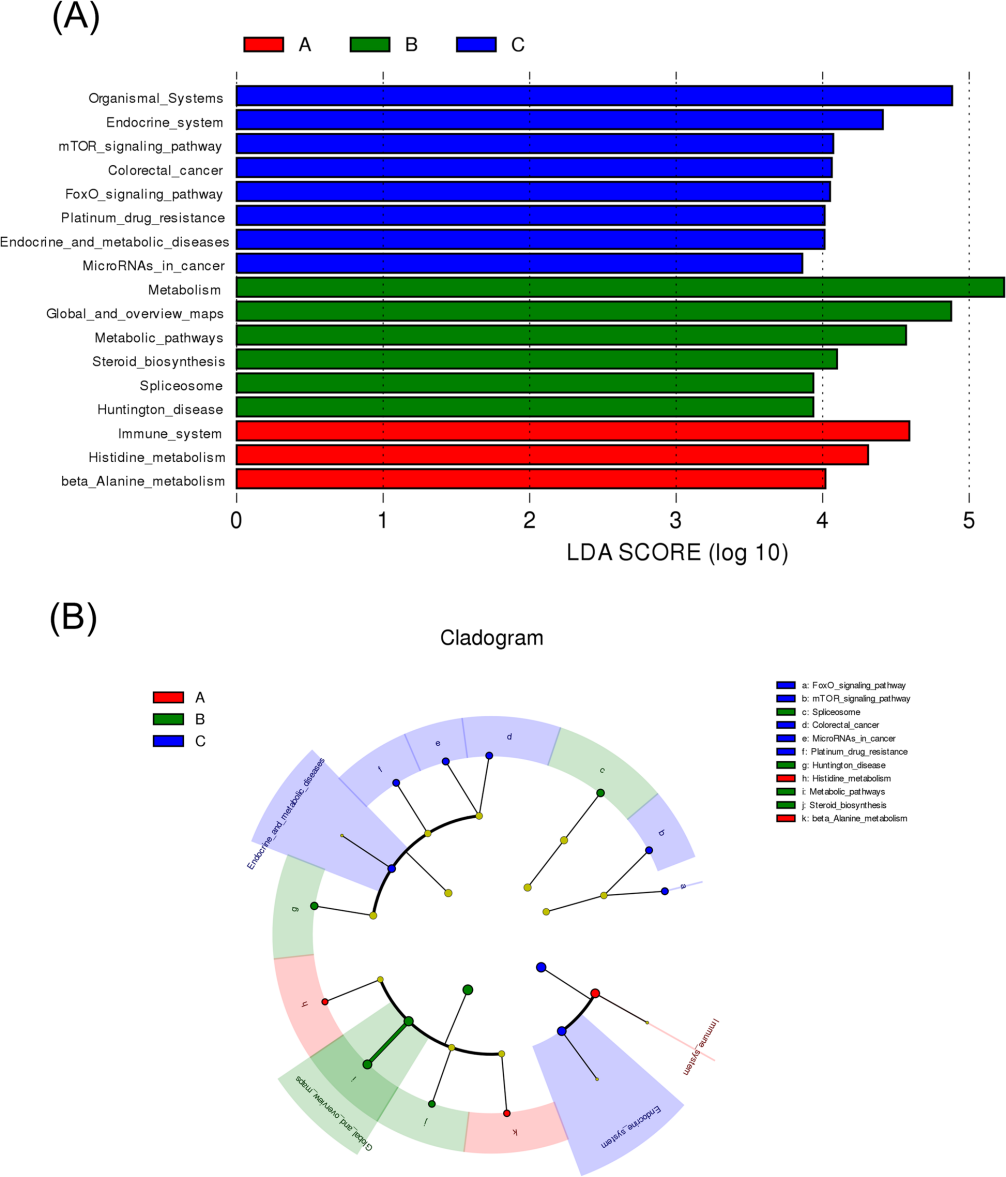
Cladogram and LDA score analysis of functions among three groups

 The potential functions of the uterine microbiota were analyzed using LEFSE (Figs. 6 and 7). They were enriched in NOD-like receptors, and IL-17 signaling pathways, which are involved in regulating various physiological processes in the uterus, such as immune response and metabolism [33, 34]. Compared to that of group A, enrichment in group B was concentrated among the metabolic, AMPK, mRNA surveillance, and Hippo signaling pathways, among which the AMPK and Hippo pathways were associated with ovulation. Group C was enriched in human diseases and cancers, suggesting that postpartum mouse uterine microorganisms are more likely to induce disease. In the comparison of the three groups (Fig. 8), group A transformed the immune system, histamine metabolism, and propionate metabolism. Group B transformed the metabolic pathways, steroid biosynthesis, and the AMPK signaling pathway. Group C intervention altered the endocrine system, Apelin and FoxO signaling pathways, colorectal cancer, microRNAs in cancer, endocrine and metabolic diseases, and platinum drug resistance.

### Validation of bacteria by qRT-PCR analysis

**Fig. 9:**
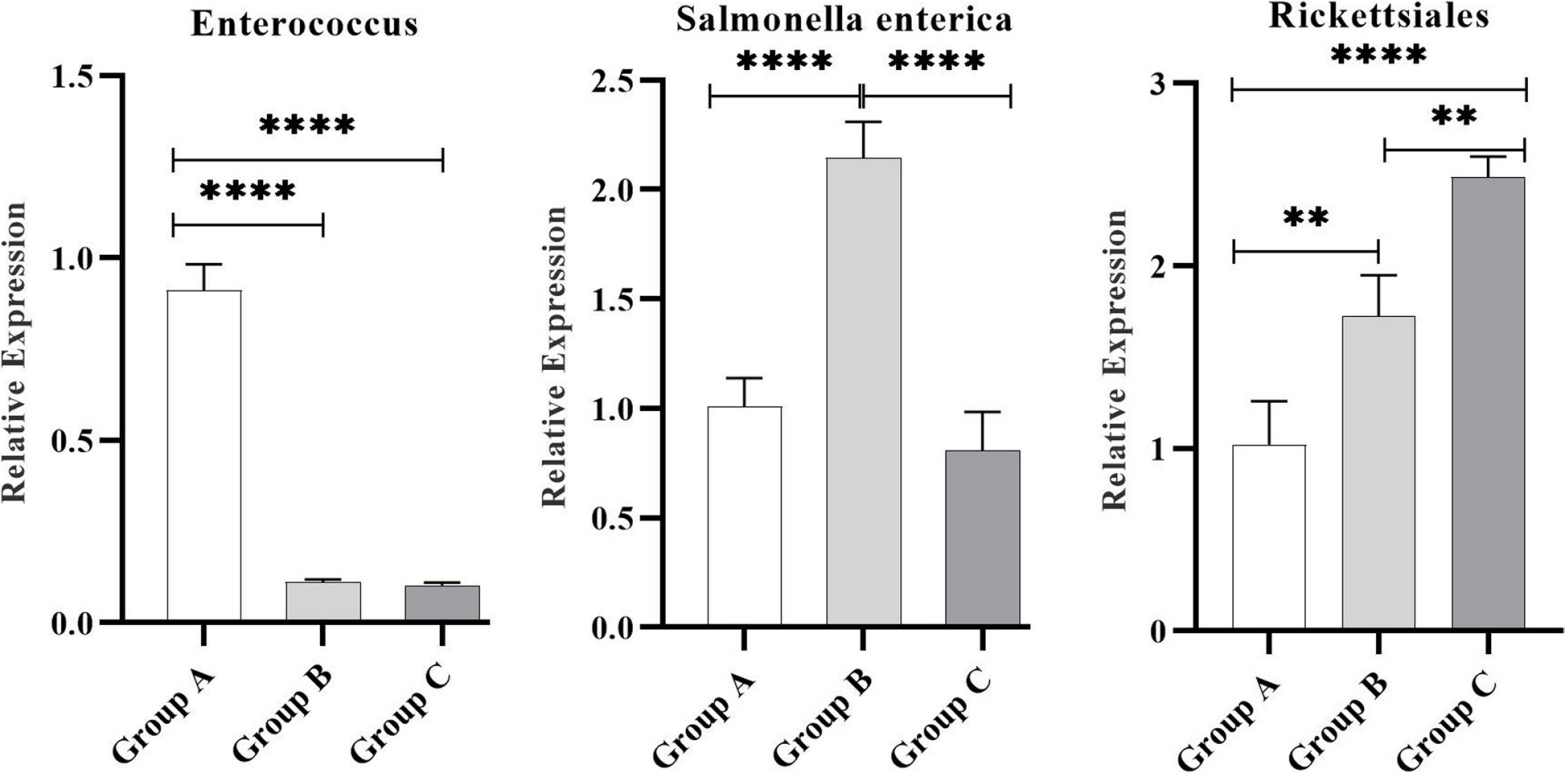
The mRNA expression of the marker gene of the highest content bacteria in the three grou ps of endometrial fluid

 For validation, the marker genes of the bacteria with the highest content in the three groups were verified by qRT-PCR analysis at the mRNA level. Enterococcus had the highest expression levels in Group A, and Salmonella enterica had the highest expression in Group B. Rickettsiales had the highest expression levels in Group C (Fig. 9). Our validation study showed that all the qRT-PCR results were consistent with the results of macrogenomic analysis.

## Discussion

**Fig. 10:**
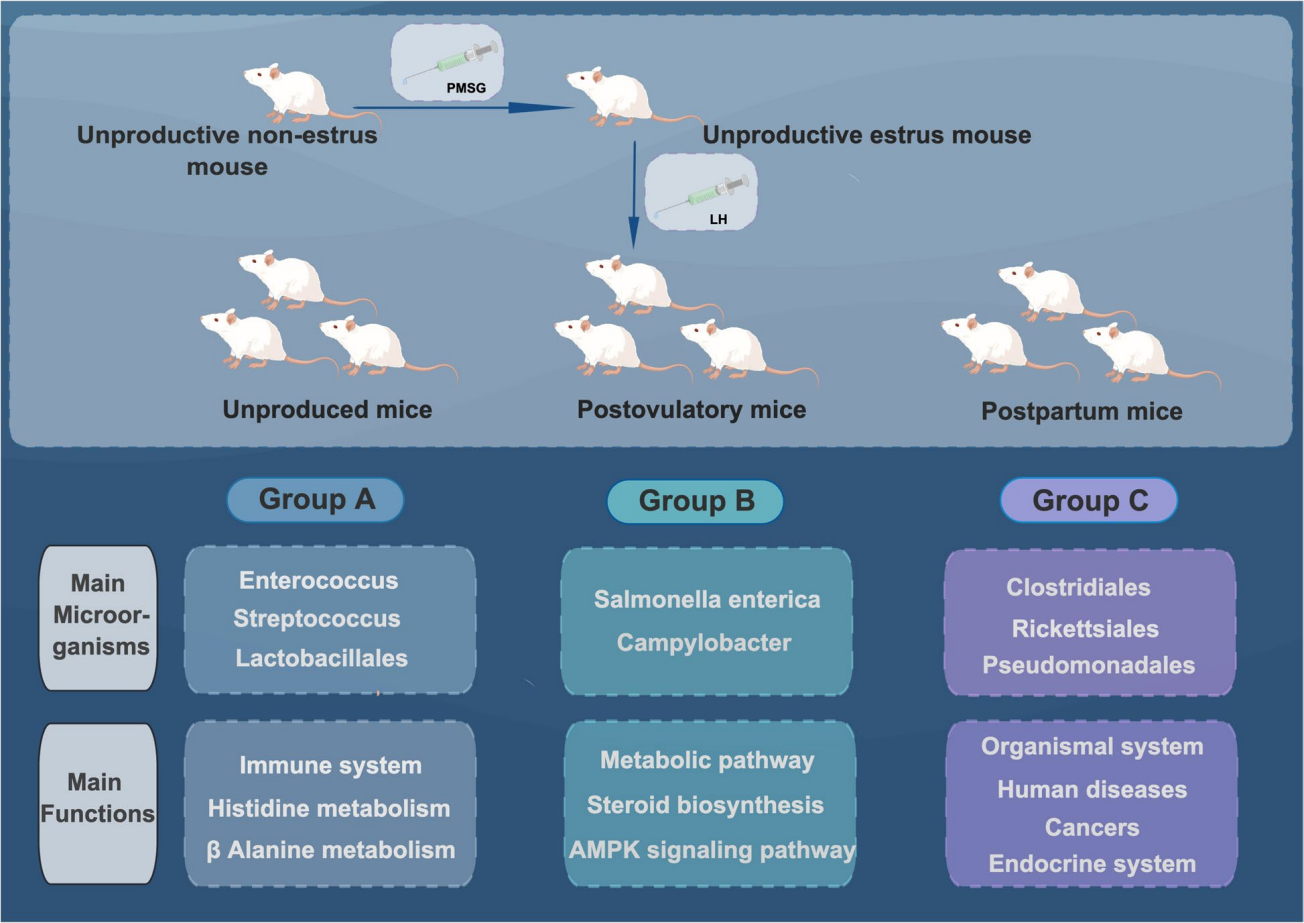
Microbial colonization and functional enrichment in mice endometrium under different reproductive st ates

 Mouse reproductive function depends on endometrial health, however, its microbial relevance to the genital tract has not been adequately studied. This study is the first to explore the microbial diversity of the uterus in different reproductive stages of animals and analyze the impact of microbial community alterations on reproductive health. The taxa in the endometrial samples collected from unproductive, post-ovulatory, and postpartum mice were distributed among 23 bacterial phyla, including the predominant phyla *Proteobacteria* and *Fimicutes* (Fig. 3A). Postovulatory mice were mainly enriched for *Salmonella enterica* and *Campylobacter* (Fig. 10); however, *Chlamydia* was detected, and *Chlamydia* infection may be associated with ovarian dysfunction due to reproductive hormones [35, 36]. In the present study, PMSG and LH were used to regulate estrus and ovulation in mice. The abundance of *Chlamydia* in the post-ovulatory group was higher than that in the unproductive group, indicating that changes in reproductive tract microbial diversity during superovulation and the induction of reproductive health diseases cannot be ignored. Pathogenic bacteria such as *Chlamydia abortus, Pseudomonas pseudomonadales, Klebsiella pneumoniae*, and *Acinetobacter baumannii* were enriched in postpartum mice, which have a greater risk of disease or infertility due to low immunity. In general, these results suggest that the exploration of microbial diversity within the intrauterine environment in various reproductive stages is instructive for analyzing the association between microbes and reproductive regulation.

The diversity of the vaginal microflora is higher in unproductive healthy women [3, 9, 37, 38], whereas it is relatively stable and homogeneous in post-ovulatory women [39, 40]. Our results showed that the microbial diversity of unproductive mice was higher than that of postovulatory and postpartum mice, confirming that there were differences in microbial diversity between the prenatal and postnatal uteri of the mice. Changes in microbial diversity in the reproductive tract of postpartum mammals indicate that parturition increases the risk of microbial infections.

Our analysis showed that supernumerary ovulation using reproductive hormones altered microbial diversity in the uterus. Comparative analysis of the three groups of mice showed that changes in microbial populations led to alterations in metabolism, biosynthesis, ovulation regulation, and reproductive system functions. This is the first study to report the use of reproductive hormones to alter the microflora of the uterus during superovulation. PMSG and LH altered microbial diversity and decreased the number of beneficial bacteria in the mouse uterus. Changes in female estrogen levels cause microbial fluctuations in the vagina and gut [41–44] and elevated estrogen levels can enhance the adhesion and colonization abilities of vaginal *Candida albicans* and *Escherichia coli* [16, 18]. Based on the above results, we concluded that reproductive hormones alter the microbial diversity of the reproductive system and are involved in the regulation of animal reproduction.

In terms of reproductive regulation, we found that mice undergoing supernumerary ovulation are more likely to harbor pathogenicity. Compared to postovulatory mice, the percentage of pathogenic microorganisms was higher in postpartum mice, which were mainly enriched in disease-related signaling pathways, whereas postovulatory mice were enriched in ovulatory functions. In terms of reproductive tract health, the microorganisms in unproductive mice were mainly enriched in pathways that maintain uterine ecological homeostasis, whereas those in postpartum mice were enriched in disease pathways. Postpartum immunity declines in women, and conditionally pathogenic bacteria in the reproductive tract become dominant (e.g., *Chlamydia, Enterococcus, Streptococcus*) [45, 46], more easily triggering diseases such as endometritis and chorioamnionitis [47, 48]. This suggests that the reproductive tract of postpartum animals is more prone to inducing pathogenic microbial infections, and that females should pay attention to the prevention of conditionally pathogenic bacteria such as *Chlamydia, Enterococcus*, and *Streptococcus* after parturition.

Overall, there were differences in the characteristics of microbial diversity in the different reproductive states. Reproductive regulation and delivery change the microbial diversity of the intrauterine environment, allowing the dominant flora to participate in the regulation of animal reproduction through metabolic pathways and steroid biosynthesis, suggesting that reproductive diseases induced by altered microbial diversity during the regulation of animal reproduction cannot be ignored.

## Data Availability

The raw sequence data have been deposited in NCBI short read archive (SRA) under BioProject ID PRJNA970465. Further inquiries can be directed to the corresponding author.
